# Preparation and Biocompatibility of Core-Shell Microspheres for Sequential, Sustained Release of BMP-2 and VEGF

**DOI:** 10.1155/2022/4072975

**Published:** 2022-11-25

**Authors:** Zheng Liu, Zhenchao Xu, Xiyang Wang, Yilu Zhang, Yunqi Wu, Dingyu Jiang, Runze Jia

**Affiliations:** ^1^Department of Orthopedics, Hunan Children's Hospital, 86# Ziyuan Road, Changsha, Hunan 410007, China; ^2^Department of Spine Surgery and Orthopaedics, Xiangya Hospital of Central South University, 87# Xiangya Road, Changsha, Hunan 410008, China; ^3^Hunan Engineering Laboratory of Advanced Artificial Osteo-Materials, 87# Xiangya Road, Changsha, Hunan 410008, China

## Abstract

Bone defect repair remains a challenge in orthopedics. This study describes the development and potential effectiveness of vascular endothelial growth factor (VEGF)/bone morphogenetic protein-2 (BMP-2) shell-core microspheres for promoting bone regeneration. Poly(L-lactic acid)/polylactic-co-glycolic acid (PLLA/PLGA) core-shell microspheres loaded with VEGF and BMP-2 were prepared by a coaxial electrospray technique, and their surface morphology, core-shell distribution, and particle size were examined. Different groups of microspheres were prepared with different placement of the growth factors, and the encapsulation efficiency and in vitro release curves were measured. Additionally, the effects of the different groups of microspheres on the proliferation and differentiation of osteoblasts and vascular endothelial cells were investigated. The prepared microspheres had a core-shell structure with good homogeneity and dispersion, a clear boundary, and a smooth surface. On scanning electron microscopy, the mean diameter of the microspheres was similar for all six preparations (*P* > 0.05). During in vitro release, growth factor was initially released via a brief burst release from the outer shell of the microsphere followed by a slower sustained release. The release of growth factors from the inner core remained relatively slow and sustained. Sequential release of different growth factors was achieved through the inconsistent release rates from the microsphere shell and inner core. All groups of microspheres showed no cytotoxicity, good biocompatibility, and the ability to promote osteoblast proliferation. The microspheres loaded with BMP-2 also promoted osteoblast differentiation, and VEGF-loaded microspheres promoted the proliferation and differentiation of vascular endothelial cells. The BMP-2 (core)/VEGF (shell) microsphere group best promoted osteoblast differentiation. The microspheres prepared in this study exhibited slow sequential release of BMP-2 and VEGF and showed good biocompatibility along with the ability to promote osteoblast differentiation and vascular endothelial cell proliferation.

## 1. Introduction

Bone defect repair continues to be a major challenge in the field of orthopedics. Growth factors can promote bone regeneration and have been used in clinical practice. However, local direct administration of growth factors results in a short half-life, easy inactivation, and rapid dilution and metabolism. Therefore, strategies for prolonging the action of growth factors in the body and maintaining appropriate drug concentrations have become a research focus. The development of drug delivery technology has produced degradable drug carriers and controllable delayed drug release systems that can prolong the action time and improve drug concentration in local lesions [1, 2]. Within the complex and highly coordinated process of natural bone development and defect repair, angiogenesis is a necessary condition for bone morphogenesis [3]. Accordingly, biomaterials that simulate appropriately timed osteogenesis and angiogenesis are needed to promote the repair of bone defects.

Bone morphogenetic protein-2 (BMP-2) possesses favorable osteoinductive activity and plays an essential regulatory role in the process of normal osteogenic differentiation. Nevertheless, bone defect repair is a complex physiological process involving coregulation of various cytokines. Multiple studies have indicated that vascular invasion plays an important role in bone injury repair and regeneration [4, 5]. Vascular endothelial growth factor (VEGF) is the main growth factor that promotes angiogenesis in vivo. VEGF promotes the proliferation and differentiation of vascular endothelial cells (VECs) and regulates the process of angiogenesis. Thus, BMP-2 and VEGF both play critical roles in osteogenesis and have complementary biological functions that can promote bone formation. Thus, their combined use in bone tissue engineering approaches may be more beneficial than the use of either factor alone.

To date, several types of encapsulation technologies have been developed for realizing the timed release of different growth factors [6–8]. Considering their application for multipurpose drug delivery and easy integration into many scaffold matrices, core-shell microspheres are an attractive option for sequential delivery of growth factors. In regenerative medicine, particle size is an important issue in clinical applications [9]. The inherent disadvantage of large spheres is the potential retention within scaffolds, especially for spheres with a complex porous three-dimensional structure, resulting in the loss of a uniform distribution of the encapsulated factors in the matrix or tissue. However, very small nanospheres are easily engulfed by surrounding cells, which limits their residence time in the implantation area and weakens their biological effects [10]. Considering the above-mentioned issues, we speculated that micron-sized core-shell spheres might not only be a favorable choice for sustained and timely release of factors that promote osteogenesis and angiogenesis but also could achieve synergistic effects via simulation of a synchronous balance of angiogenesis and osteogenesis in the process of bone formation.

For clinical application, to avoid the potential shortcomings of autologous bone and allogeneic bone, bioactive bone repair materials have been actively pursued. The key strategy for designing biomaterials for bone repair is to simulate the natural bone healing process. In the present study, poly(L-lactic acid)/polylactic-co-glycolic acid (PLLA/PLGA) microspheres loaded with VEGF and BMP-2 were prepared by the coaxial electrospray method to achieve sequential, sustained growth factor release for the ultimate purpose of simulating the bone repair process and promoting the formation of new blood vessels within new bone. The prepared microspheres were characterized, and their effects on the proliferation and differentiation of osteoblasts and VECs were investigated.

## 2. Materials and Methods

### 2.1. Materials

PLGA, PLLA, BMP-2, VEGF165, sodium *β*-glycerophosphate, ascorbic acid, and coumarin-6 were purchased from Sigma-Aldrich (St. Louis, MO, USA). The MC3T3-E1 Subclone-14 osteogenic precursor cell line and human umbilical vein endothelial cell line EA.HY926 were obtained from Cell Resource Center, Shanghai Institutes of Biological Sciences, Chinese Academy of Sciences. Alpha Minimal Essential Medium (*α*-MEM), high-glucose Dulbecco's Modified Eagle Medium, fetal bovine serum, double antibiotic solution (streptomycin and penicillin), and 0.25% trypsin were purchased from Hyclone (Logan, UT, USA). Osteopontin (OPN) antibody, *β*-actin antibody, runt-related transcription factor 2 (RUNX2) antibody, horseradish peroxidase- (HRP-) labeled goat anti-rabbit IgG, and HRP-labeled goat anti-mouse IgG were bought from Proteintech (Rosemont, IL, USA). Enzyme-linked immunosorbent assay (ELISA) kits for BMP-2 and VEGF were obtained from Cusabio Biotech Co., Ltd. (Wuhan, Hubei, China). A CCK-8 test kit, live/dead cell staining kit, alkaline phosphatase (ALP) detection kit, ALP staining kit, and nitric oxide (NO) detection kit were purchased from Shanghai Beyotime Biotechnology Co., Ltd. (Shanghai, China). Alizarin red dye and a kit for reverse transcription polymerase chain reaction were obtained from Beijing ComWin Biotech Co., Ltd. (Beijing, China).

**Figure 1:**
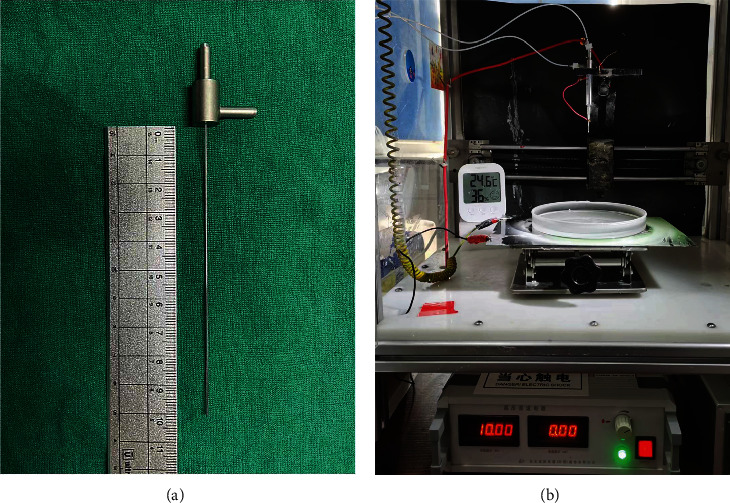
Coaxial electrostatic spray experimental device: (a) coaxial needle and (b) electrostatic spinning machine.

The coaxial electrospray device consisted mainly of an electrostatic spinning machine and a coaxial needle, as shown in Figure 1. The internal and external diameters of the outer tube of the coaxial needle were 0.50 mm and 0.72 mm, respectively, and those of the inner tube were 0.20 mm and 0.40 mm, respectively.

### 2.2. Preparation of Microspheres

Six microsphere formulations were prepared: (1) BMP-2 in the core and VEGF in the shell (BMP-2/VEGF group); (2) BMP-2 in the core and no factor in the shell (BMP-2/blank group); (3) no factor in the core and VEGF in the shell (blank/VEGF group); (4) two factors, BMP-2 and VEGF, in both the nucleus and shell (double/double group); (5) VEGF in the core and BMP-2 in the shell (VEGF/BMP-2 group); (6) no factor in either the core or shell (blank/blank group).

The preparation solutions for blank microspheres consisted of 500 mg PLGA (molecular weight 40,000 Da) in 10 ml dichloromethane (DCM) and 500 mg PLLA (molecular weight 90,000 Da) in 10 ml ethyl acetate (EA). The preparation solutions for factor-loaded microspheres consisted of VEGF in deionized water (10 *μ*g/ml), BMP-2 in deionized water (100 *μ*g/ml), PLGA in EA (5%, w/v), and PLLA in DCM (5%, w/v). The growth factor solution served as the aqueous phase and the polymer solution as the organic phase. The aqueous and organic solutions were mixed at a volume ratio of 1 : 10 and placed in an ultrasonic shaker until a homogeneous emulsion was formed. The details for each microsphere preparation are shown in Table 1.

The polymer concentration was set at 5%; the flow rates of the solutions in the inner and outer tubes of the coaxial needle were set at 1.5 ml/h, and the voltage was set at 10.0 kV. The injection pumps for the PLGA and PLLA solutions were connected to the outer needle tube and the inner needle tube of the coaxial needle, respectively. The electrospinning machine was initiated, and the voltage was adjusted progressively to 10.0 kV. As the voltage was increased, the solution leaving the tip of the coaxial needle changed from a droplet shape to a linear shape, then finally to mist-like droplets, and ultimately a stable Taylor cone. The microspheres were collected about 15 cm below the coaxial needle tip in a glass vessel filled with an appropriate amount of absolute ethanol. The collected anhydrous alcohol microsphere suspension was transferred to a 50 ml centrifugal tube, centrifuged for 3 min at 175 × g, and then washed with double-steamed water three times after removal of the ethanol from the upper layer. Next, the solution was incubated at -80°C for 30 min, freeze-dried for 72 h, and stored in the refrigerator at 4°C away from light for use.

### 2.3. Observation of Core-Shell Microsphere Structure

For observation of core-shell microsphere structure under confocal laser scanning microscopy (CLSM), microspheres were prepared with a trace of coumarin-6 added in the PLLA/DCM solution to dye this layer light green. For observation of the surface morphology and particle size of the core-shell microspheres by scanning electron microscopy (SEM), freeze-dried microspheres were sprayed with gold for 60 sec and then observed. For the measurement of microsphere diameter, two fields of view were randomly selected for each group of microspheres, and 10 microspheres were randomly selected in each field for diameter measurement.

### 2.4. Determination of Growth Factor Encapsulation Efficiency in Core-Shell Microspheres

Three samples of each microsphere formulation were weighed, and 20 mg of each sample was dissolved in 1 ml DCM by ultrasonic vibration at low temperature. After the core-shell structural polymers PLGA and PLLA were completely dissolved, 1 ml phosphate-buffered saline (PBS) was added. This solution was vortexed for 10 min and then placed in a low-temperature high-speed centrifuge at 34400 × g for 3 min. After centrifugation, the aqueous layer was carefully absorbed. This procedure was repeated twice, and then the VEGF and BMP-2 concentrations in the recovered solutions were determined by ELISA. The encapsulation efficiency was calculated as follows: Encapsulation efficiency = (Actual total amount of growth factors in the sample/Theoretical total amount) × 100%.

### 2.5. Measurement of In Vitro Growth Factor Release from Core-Shell Microspheres

Samples consisting of 20 mg microspheres were placed in a dialysis bag and then in a centrifuge tube containing 10.0 ml PBS. The centrifuge tube was then placed on a constant temperature (37°C) orbital shaker at 120 rpm. At the indicated time points (1, 2, 4, 6, 8, 11, 14, 18, 22, or 27 days), the PBS from the centrifuge tube was aspirated and immediately replaced. The recovered PBS was stored at –20°C until analysis. The concentrations of BMP-2 and VEGF in the solutions collected at each time point were measured by ELISA, and the released amounts were calculated for preparation of release curves.

### 2.6. In Vitro Evaluation of Core-Shell Microsphere Biocompatibility

#### 2.6.1. Cell Culture

MC3T3-E1 cells were cultured in *α*-MEM containing 10% fetal bovine serum and 1% double antibiotic solution (100 U/ml of both streptomycin and penicillin) in a cell incubator at 37°C with 5% CO_2_, and the medium was changed every 2 days. The cells were passaged at 80%–90% confluency and subcultured to the P3 generation for use in experiments.

#### 2.6.2. Exposure of Cells to Microspheres

P3 generation MC3T3-E1 cells at a concentration of 2 × 10^4^ cells/ml in 1 ml medium were added to the lower chamber of a 24-well Transwell plate, and 20 mg of the specified core-shell microsphere formulation was placed in the upper chamber. For the control group, the wells were seeded with the same number of cells, but no microspheres were added to these wells. Three wells were prepared for each group. The solution was changed every 2 days.

#### 2.6.3. Measurement of Osteoblast Proliferation upon Exposure to Factor-Loaded Core-Shell Microspheres

After 1, 3, 5, and 7 days in culture with the microspheres, 50 *μ*l CCK-8 solution was added to each well for further incubation for 4 h. The supernatant from each well was then carefully collected and transferred to the wells of a 96-well plate. The amount of solution was adjusted to 100 *μ*l/well, and the optical density (OD) value was detected at 450 nm wavelength by a microplate reader.

#### 2.6.4. Measurement of Osteoblast Activity upon Exposure to Factor-Loaded Core-Shell Microspheres

After 3 days in culture with the microspheres, the Transwell plate chambers were discarded, the medium was aspirated, and the cells were washed three times with PBS. The prepared live/dead cell staining working solution (10 ml with 8 *μ*M propidium iodide and 2 *μ*M calcein AM) was added for incubation at room temperature for 45 min. After removal of the staining solution, a trace of PBS was added, and the labeled cells were observed under a fluorescence microscope.

### 2.7. Evaluation of the Effects of Factor-Loaded Core-Shell Microspheres on Osteoblast Differentiation

#### 2.7.1. Detection of ALP Activity

Osteogenic induction medium (100 ml *α*-MEM +316 mg sodium *β*-glycerophosphate +5 mg ascorbic acid +5 *μ*L dexamethasone) was used to induce differentiation of MC3T3-E1 cells cultured with the different core-shell microsphere formulations. The medium was changed every 3 days. After the 12th day of osteogenesis induction, ALP activity was measured using two methods. Some cell samples were washed with PBS three times and fixed with 4% paraformaldehyde for 2 min. Then, the cell fixation solution was removed, and an appropriate amount of ALP staining working solution was added. The cells were incubated in darkness at room temperature for 20 min and observed under an inverted microscope. Other cell samples collected on the 12th day of osteogenic induction were used for detection of ALP activity by an enzyme labeling method. The collected cells were fully lysed with cell lysis buffer, washed three times with PBS, and centrifuged at 25275 × g for 10 min. ALP activity was quantified using an ALP assay kit.

#### 2.7.2. Alizarin Red Staining

On the 14th day of osteogenic induction, cells in each group were collected, washed twice with PBS, and fixed with 4% paraformaldehyde for 30 min at room temperature. After removal of the cell fixation solution, the cells were washed with PBS for three times. Then, the cells were stained with Alizarin red for 5 min and washed with PBS for three times. The stained cells were observed under an inverted microscope.

#### 2.7.3. Detection of Osteogenesis-Related Gene Expression by Real-Time PCR

Cells were collected after 14 days of osteogenic induction. The total RNA of each group was extracted and reverse transcribed into cDNA. The mRNA expression levels of RUNX2, osteocalcin (OCN), and OPN were detected by real-time quantitative PCR using the primer sequences listed in Table 2.

The PCR cycling parameters were set as follows: predenaturation at 95°C for 10 min, denaturation at 95°C for 15 s, and annealing at 60°C for 30 s for 40 cycles. The final extension step was conducted at 72°C for 10 min. The end products were subjected to 1.5% agarose gel electrophoresis, and actin was used as the internal reference gene.

#### 2.7.4. Detection of Osteogenesis-Related Protein Expression by Western Blotting

Cells were collected after 21 days of osteogenic induction. The protein concentration was determined by the BCA standard curve method. For western blot analysis of protein expression, the proteins were subjected to gel electrophoresis followed by transfer to nitrocellulose membranes. The membranes were then incubated overnight at 4°C with RUNX2, OPN, and *β*-actin primary antibodies at a dilution ratio of 1 : 1000. The next day, the membranes were incubated with secondary antibodies at a dilution ratio of 1 : 10000 at room temperature for 2 h, before being three 10 min washes with Tris-buffered saline containing Tween 20 (TBST) solution. Enhanced chemiluminescence solution was added to the washed samples, incubated for 1 min, and developed for detection of the absorbance and molecular weight of labeled proteins.

### 2.8. Evaluation of the Effects of Factor-Loaded Core-Shell Microspheres on VECs

#### 2.8.1. Measurement of VEC Proliferation, Activity, and NO Secretion

EA.hy926 cells of the P3 generation were exposed to different formulations of microspheres following the same Transwell culture method used for osteoblasts. The proliferation and activity of VECs were evaluated using the same CCK-8 detection and live/dead staining methods used for osteoblasts. The amount of NO secretion was detected using a NO detection kit according to the manufacturer's instructions.

#### 2.8.2. Detection of Angiogenesis-Related Gene Expression by Real-Time PCR

Expressions of the angiogenesis-related genes VEGF and hypoxia-inducible factor 1 alpha (HIF-1*α*) were detected by real-time quantitative PCR using the primer sequences listed in Table 3.

### 2.9. Statistical Analysis

SPSS 24.0 software was used for all statistical analyses, and the data are expressed as mean ± standard deviation. One-way analysis of variance was performed to identify differences among the groups. Differences were considered significant if *P* values were less than 0.05.

## 3. Results

### 3.1. Structure and Size of the PLLA/PGLA Core-Shell Microspheres

**Figure 2:**
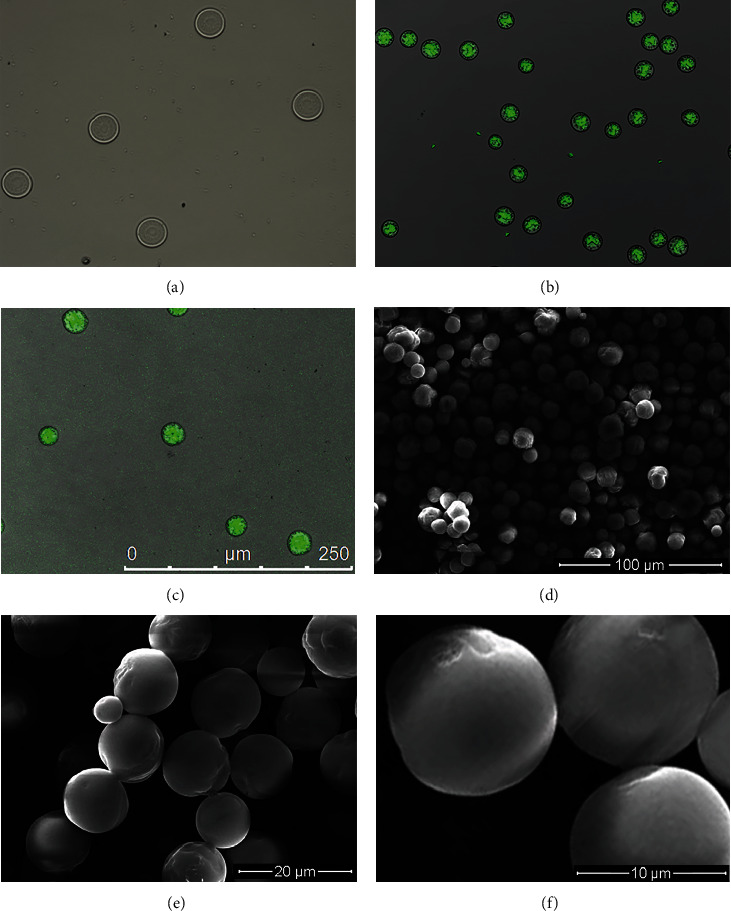
Representative images of core-shell microspheres taken by (a) optical microscopy, (b, c) CLSM, and (d–f) SEM.

Under optical microscopy, the microspheres exhibited an obvious core-shell structure, round shape, and good uniformity. Under CLSM, the morphology of the microspheres was found to be a clear and obvious core-shell structure. Under SEM, the microspheres had a regular morphology, strong stereoscopic impressions, round and smooth surface, and no obvious particle–particle adhesion (Figure 2).

As measured in SEM images, the mean diameter of the microspheres was similar for all six preparations (*P* > 0.05). The mean microsphere diameters were 17.85 ± 1.45 *μ*m for the BMP-2 (core)/VEGF (shell) group, 17.52 ± 1.06 *μ*m for the BMP-2/blank group, 17.16 ± 1.10 *μ*m for the blank/VEGF group, 17.58 ± 1.08 *μ*m for the double/double group, 17.36 ± 1.32 *μ*m for the VEGF/BMP-2 group, and 17.66 ± 1.54 *μ*m for the blank/blank group.

### 3.2. Growth Factor Encapsulation in Core-Shell Microspheres

The encapsulation efficiency of the microsphere preparation process was evaluated using ELISA kits to measure the concentrations of growth factors contained within the dissolved microspheres. The encapsulation efficiency was calculated by dividing the amount of recovered growth factor by the amount used in the microsphere preparation. Some variation was observed in the encapsulation efficiency among the different formulations, and this can be attributed to the different locations of the growth factors in different groups of microspheres (Table 4).

### 3.3. Sustained Growth Factor Release from Core-Shell Microspheres In Vitro

**Figure 3:**
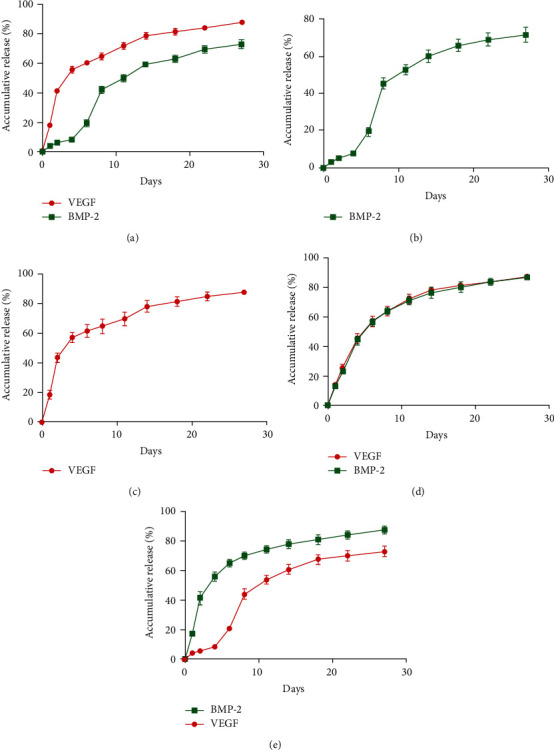
Release curves for BMP-2 and VEGF release from the different core-shell microspheres: (a) BMP-2/VEGF group; (b) BMP-2/blank group; (c) blank/VEGF group; (d) double/double group; (e) VEGF/BMP-2 group.

Figure 3 shows the growth factor release curves for the five groups of growth factor-loaded microspheres. The corresponding cumulative release kinetics showed that sequential release of two growth factors was achieved in the BMP-2/VEGF and VEGF/BMP-2 groups. The growth factor in the shell was released initially in a short burst and then gradually at a sustained level, while the release of growth factor from the core was always relatively steady. Overall, sequential release of different growth factors was achieved through the inconsistent release rates of the shell and core materials.

### 3.4. Effects of Growth Factor-Loaded Core-Shell Microspheres on Osteoblast Viability

**Figure 4:**
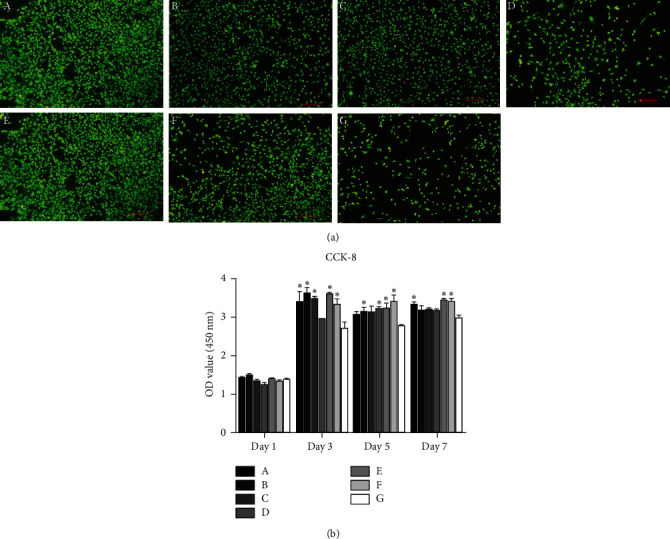
(a) Live/dead staining of osteoblasts after 3 days of exposure to the different formulations of core-shell microspheres. Magnification, ×40; (b) proliferation of osteoblasts exposed to the different formations of core-shell microspheres according to the CCK-8 assay, ^∗^*P* < 0.05 compared with the control group. (A) BMP-2/VEGF group; (B) BMP-2/blank group; (C) blank/VEGF group; (D) double/double group; (E) VEGF/BMP-2 group; (F) blank/blank group; (G) control group (no microspheres).

Live/dead staining showed no obvious dead cells in any group after exposure to microspheres for 3 days. Thus, none of the microsphere formulations was toxic to the cells, indicating their good biocompatibility. In agreement with the proliferation assay results, the double/double and control groups had lower cell numbers at this time point, and no connections were observed between these cells. The BMP-2/blank, blank/VEGF, and blank/blank groups had more cells, with some connections observed between cells. The BMP-2/VEGF and VEGF/BMP-2 groups had the highest numbers of cells, with abundant connections and spreading observed in these groups (Figure 4(a)).

As shown in Figure 4(b), after 1 day of exposure of the different formulations of microspheres, no difference in the number of cells was observed among the groups (*P* > 0.05). After 3 days of exposure to the microspheres, the number of cells in each group had increased significantly. The BMP-2/VEGF, BMP-2/blank, blank/VEGF, VEGF/BMP-2, and blank/blank groups showed higher cell numbers than the control group not exposed to microspheres (*P* < 0.05), and the mean numbers of cells in the double/double and control groups showed no significant difference. After 5 days in culture, the BMP-2/blank, double/double, VEGF/BMP-2, and blank/blank groups showed higher cell numbers than the control group (*P* < 0.05), and no significant difference was observed among the BMP-2/VEGF, blank/VEGF, and control groups (*P* > 0.05). After 7 days in culture, the BMP-2/VEGF, VEGF/BMP-2, and blank/blank groups had higher cell numbers than the control group (*P* < 0.05), while no significant difference was observed among the BMP-2/blank, blank/VEGF, double/double, and control groups (*P* > 0.05).

### 3.5. Effects of Growth Factor-Loaded Core-Shell Microspheres on Osteoblast Differentiation

**Figure 5:**
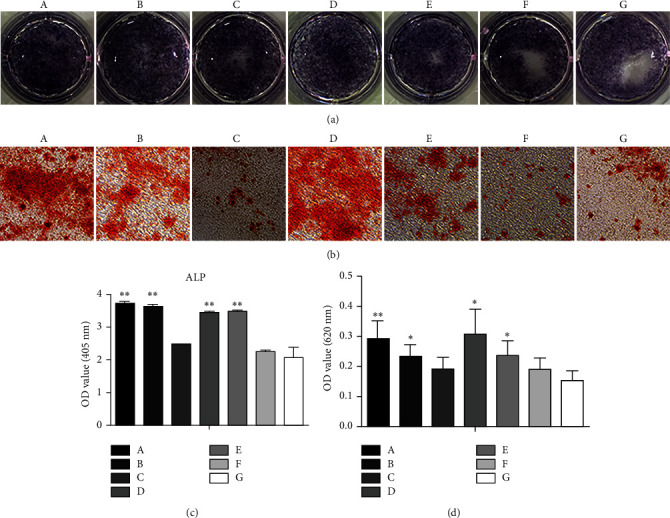
(a) ALP staining in cultures of osteoblasts exposed to the different formulations of core-shell microspheres. (b) Alizarin red staining in osteoblasts exposed to the different formulations of core-shell microspheres. (c) Quantitative results for ALP production by osteoblasts exposed to the different formulations of core-shell microspheres, ^∗∗^*P* < 0.01 compared with the control group. (d) Quantitative results for Alizarin red staining in osteoblasts exposed to the different formulations of core-shell microspheres, ^∗^*P* < 0.05 and ^∗∗^*P* < 0.01 compared with the control group. (A) BMP-2/VEGF group; (B) BMP-2/blank group; (C) blank/VEGF group; (D) double/double group; (E) VEGF/BMP-2 group; (F) blank/blank group; (G) control group (no microspheres).

Compared with the blank/blank and no microsphere control groups, ALP staining was visibly increased in cells exposed to microspheres loaded with BMP-2, including the BMP-2/VEGF, BMP-2/blank, double/double, and VEGF/BMP-2 groups, and the staining intensity was darker for the BMP-2/VEGF group than for the other groups (Figure 5(a)). The results for ALP quantification are depicted in Figure 5(c). Osteoblasts exposed to the BMP-2/VEGF, BMP-2/blank, double/double, and VEGF/BMP-2 microspheres produced significantly more ALP than those exposed to the blank control microspheres (*P* < 0.05), and ALP production did not differ significantly among the blank/VEGF, blank/blank, and control groups (*P* > 0.05).

Alizarin red staining was more apparent in the BMP-2/VEGF, BMP-2/blank, double/double, and VEGF/BMP-2 groups than in the no microsphere control group. Alizarin red staining showed that the content of calcium nodules in these groups was higher than that in the control group, but no significant difference was observed among the blank/VEGF, blank/blank, and control groups (Figure 5(b)). Quantitative analysis of alizarin red staining showed that the content of calcium nodules in the BMP-2/VEGF group was significantly higher than that in the no microsphere control group (*P* < 0.01), and the content of calcium nodules was also higher in the BMP-2/blank, double/double, and VEGF/BMP-2 groups than that in the control group (*P* < 0.05; Figure 5(d)). The content of calcium nodules did not differ significantly among the blank/VEGF, blank/blank, and control groups (*P* > 0.05).

**Figure 6:**
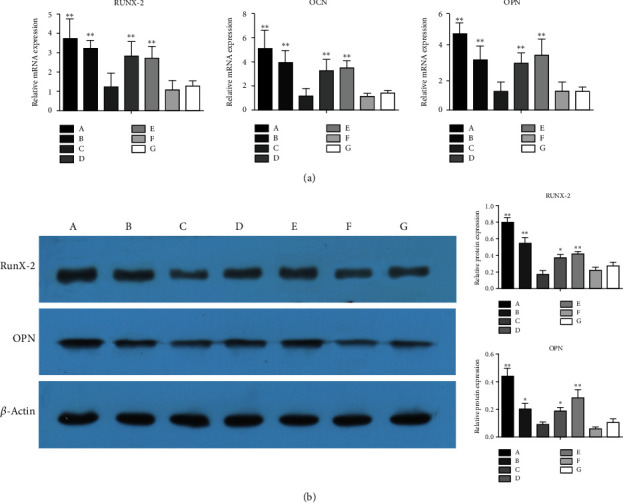
(a) mRNA expression of RUNX2, OCN, and OPN genes in osteoblasts exposed to the different formulations of core-shell microspheres, ^∗^*P* < 0.05 and ^∗∗^*P* < 0.01 compared with the control group. (b) Expression of RUNX2 and OPN proteins osteoblasts exposed to the different formulations of core-shell microspheres, ^∗^*P* < 0.05 and ^∗∗^*P* < 0.01 compared with the control group (G). (A) BMP-2/VEGF group; (B) BMP-2/blank group; (C) blank/VEGF group; (D) double/double group; (E) VEGF/BMP-2 group; (F) blank/blank group; (G) control group (no microspheres).

Osteoblasts exposed to each of the microsphere formulations loaded with BMP-2 (BMP-2/VEGF, BMP-2/blank, double/double, and VEGF/BMP-2 microspheres) expressed higher levels of osteogenic RUNX2, OCN, and OPN genes compared with cells in the control group not exposed to microspheres (*P* < 0.01), while the expression levels of these genes did not differ significantly among the blank/VEGF, blank/blank, and control groups (*P* > 0.05). Expression of the RUNX2 gene was not significantly different in the BMP-2/VEGF group compared with the BMP-2/blank, double/double, and VEGF/BMP-2 groups (*P* > 0.05). However, expression of the OCN gene was higher in the BMP-2/VEGF group than in the double/double and VEGF/BMP-2 groups (*P* < 0.05) but did not differ significantly between the BMP-2/VEGF group and the BMP-2/blank group (*P* > 0.05). Expression of the OPN gene was higher in the BMP-2/VEGF group than in the BMP-2/blank and double/double groups (*P* < 0.01) and higher than that of VEGF/BMP-2 group (*P* < 0.05; Figure 6(a)).

Consistent with the results for osteogenic gene expression, the relative expression of RUNX2 protein in cells exposed to the BMP-2–loaded microspheres was greater than that in the no microsphere control group (*P* < 0.01 for the BMP-2/VEGF, BMP-2/blank, and VEGF/BMP-2 groups and *P* < 0.05 for the double/double group). No significant difference in RUNX2 protein expression was observed among cells exposed to microspheres not loaded with BMP-2 (*P* > 0.05 for the comparison of the blank/VEGF and blank/blank groups with the control group). The relative expression of OPN protein also was greater in cells exposed to the BMP-2–loaded microspheres than in cells exposed to the BMP-2/VEGF and VEGF/BMP-2 (*P* < 0.01 for the BMP-2/VEGF and VEGF/BMP-2 groups and *P* < 0.05 for the BMP-2/blank and double/double groups), while again, no significant difference was observed between the blank/VEGF and blank/blank groups compared with the control group (*P* > 0.05). Furthermore, the protein expression levels of both RUNX2 and OPN were significantly higher in the BMP-2/VEGF group than in the BMP-2/blank, double/double, and VEGF/BMP-2 groups (*P* < 0.01; Figure 6(b)).

### 3.6. Effects of Growth Factor-Loaded Core-Shell Microspheres on VEC Viability

**Figure 7:**
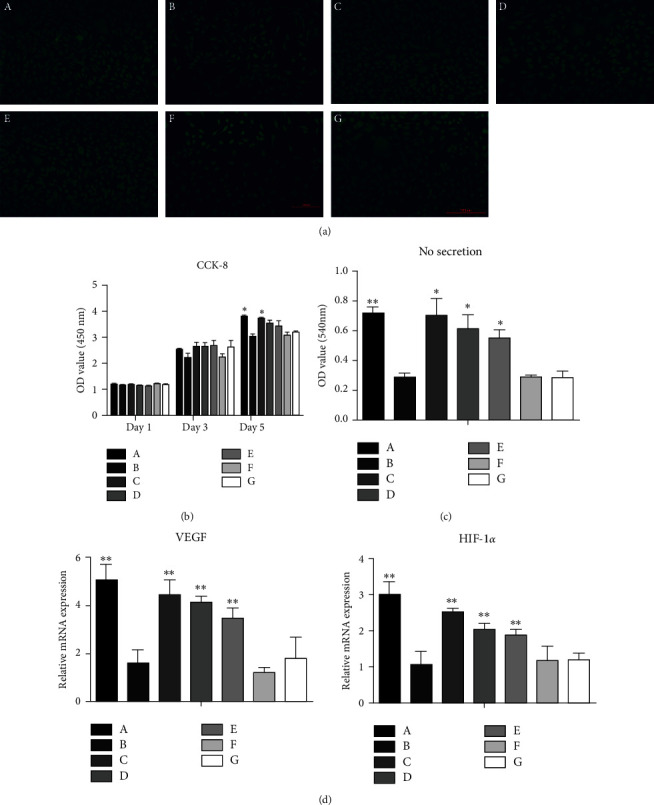
(a) Live/dead staining of vascular endothelial cells after 3 days of exposure to the different formulations of core-shell microspheres. Magnification, ×100. (b) Proliferation of vascular endothelial cells exposed to the different formations of core-shell microspheres according to the CCK-8 assay, ^∗^*P* < 0.05 compared with the control group. (c) NO secretion by vascular endothelial cells exposed to the different formulations of core-shell microspheres, ^∗^*P* < 0.05 and ^∗∗^*P* < 0.01 compared with the control group. (d) mRNA expression of VEGF and HIF-1*α* by vascular endothelial cells exposed to the different formulations of core-shell microspheres, ^∗^*P* < 0.05 and ^∗∗^*P* < 0.01. (A) BMP-2/VEGF group; (B) BMP-2/blank group; (C) blank/VEGF group; (D) double/double group; (E) VEGF/BMP-2 group; (F) blank/blank group; (G) control group (no microspheres).

Live/dead staining of VECs after exposure to microspheres in culture for 3 days showed no obvious cell death in any group, further confirming the biocompatibility of the growth factor-loaded core-shell microspheres (Figure 7(a)).

On days 1 and 3 in culture with the different microsphere formulations, the numbers of VECs showed no differences among the groups (*P* > 0.05). However, on the 5th day in culture, VEC numbers were higher in the BMP-2/VEGF and blank/VEGF groups than in the no microsphere control group (*P* < 0.05), while no significant difference in cell number continued to be observed for comparison of the BMP-2/blank, double/double, VEGF/BMP-2, and blank/blank groups with the no microsphere control group (*P* > 0.05) (Figure 7(b)).

The results for NO secretion by VECs exposed to different microspheres are shown in Figure 7(c). Significantly greater NO secretion was detected in all cell groups exposed to VEGF-loaded microspheres compared with the no microsphere control group (*P* < 0.01 for the BMP-2/VEGF group and *P* < 0.05 for the blank/VEGF, double/double, and VEGF/BMP-2 groups).

### 3.7. Effects of Growth Factor-Loaded Core-Shell Microspheres on the Angiogenic Activity of VECs

The expression levels of the angiogenesis-related VEGF and HIF-1*α* genes were significantly higher in all groups of cells exposed to VEGF-loaded microspheres (*P* < 0.01 for the BMP-2/VEGF, blank/VEGF, double/double, and VEGF/BMP-2 groups compared with the control group), while no differences in the expression levels of these genes were detected in the BMP-2/blank and blank/blank groups compared with the no microsphere control group (*P* > 0.05; Figure 7(d)).

## 4. Discussion

Despite the widespread topical use of growth factors in clinical practice, challenges remain with their delivery, due to their short half-life, easy inactivation, and easy dilution metabolism. In recent years, much research has been carried out on materials designed for the slow sustained release of growth factors, and remarkable progress has been achieved. New slow-release composite systems can slow the release rate of growth factors, thereby increasing their bioavailability.

Sustained-release composite systems have been tested for a variety of important growth factors and shown to offer synergistic release kinetics and provide a promising method for enhancing bone regeneration by simulating the cascade of spatiotemporal signals involved in natural bone development and spontaneous healing [11–13]. Core-shell microspheres specifically have demonstrated great potential for achieving sustained release of growth factors [10]. In the present study, we successfully manufactured different formulations of core-shell microspheres by a coaxial electrospray technique, in which the PLGA and PLLA portions were loaded with VEGF or BMP-2. Our results revealed that growth factor activity was well maintained during the microsphere preparation process, and sequential release of growth factors was achieved.

At present, many methods have been reported to prepare putamen microspheres, including an emulsion solvent evaporation method, electrostatic spray method, electrostatic dripping method, and self-assembly method. Coaxial electrospray has been proven to offer the advantages of maintaining the structural integrity of microspheres, reducing the particle size distribution, and maintaining the biological activity of loaded proteins [14, 15]. In the present study, two types of drugs with different physicochemical properties (water soluble or fat soluble) were successfully embedded in the outer shell and inner core of the putamen polymer microspheres by coaxial electrospray, which allows for release of the two drugs simultaneously or sequentially. By changing the relative size of the nuclei in the putamen structure, the release profiles for the two drugs can be precisely controlled. Chang et al. [16] successfully manufactured a hollow microsphere with only a shell component using coaxial electrospray, with polymethylsilsesquioxane as the model shell material encapsulating a volatile liquid perfluorohexane as the core, which was then evaporated. Another study, focusing on the control of initial release, prepared core-shell microspheres using PLGA and alginate and confirmed that high molecular weight PLGA better inhibited the initial burst release of protein compared to low molecular weight PLGA [17].

Therefore, the ability to generate particles with a core-shell structure that can continuously release two drugs via a simple fabrication method is of great significance. Toward this goal, the present study investigated the release characteristics of core-shell microspheres prepared simply by high-voltage coaxial electrospray. During the fabrication process, the major factors responsible for maintaining the stability of the microsphere core-shell structure were balanced among electrostatic repulsion, surface tension, viscoelastic force, and flow rate. In our preliminary experiments, the experimental conditions for high-voltage coaxial electrospray were explored thoroughly. The electrostatic repulsion was adjusted by changing the voltage, and a voltage of 10 kV was found to achieve core-shell microspheres with the best structure and shape. In addition, the liquid flow rates in the inner and outer tubes were set at 1.5 ml/h assuming the inner and outer tube diameters of the coaxial needle were fixed. In the present study, PLGA was dissolved in EA, and PLLA was dissolved in DCM, due to the extremely low solubility of PLLA in EA, which effectively reduced the dispersion of the solutions in the inside and outside tubes of the coaxial needle tip during high-voltage electrostatic spray [18]. Meanwhile, the concentrations of 5% PLLA solution and 5% PLGA solution had the optimal effect on core-shell microsphere preparation.

Our results indicate that the core-shell microspheres prepared by high-voltage coaxial electrospray could not only maintain the activity of growth factors but also realize the independent release of the two growth factors governed by the placement of each factor in the two-component particles. VEGF in the shell was released in the form of an initial burst release, with nearly 70% of the loaded VEGF released in the first 10 days in solution. As reported previously, VEGF secretion occurs in the early healing stage after a bone fracture, and the peak VEGF concentration appears over 5–10 days after fracture [10, 19]. Therefore, the release pattern of VEGF achieved in this study aligns with the physiological pattern. BMP-2, which plays a key role in bone regeneration, also is produced in the process of fracture healing. BMP-2 release from the microsphere core was relatively steady, rather than a burst release, which is consistent with the physiological time course of BMP-2 production during bone defect healing [8]. Long-term continuous administration can not only overcome the rapid degradation of BMP-2 in the complex local enzymatic environment but also avoid heterotopic ossification and postoperative hematoma and swelling caused by excessive local doses of BMP-2 [20]. Kempen et al. [21] reported that sustained release of BMP-2 could enhance the curative effect of ectopic osteogenesis and improve the repair of long bone defects in situ. The above evidence further supports our conclusion that the core-shell microspheres prepared in this study can be used as an effective carrier for controlled release of two growth factors at different kinetic rates. Both VEGF and BMP-2 are hydrophilic proteins and thus rapidly dissolve in PBS. During release, VEGF in the shell is in direct contact with PBS, and thus, a burst release is possible. The chemical composition of the PLGA shell provides a barrier to slow BMP-2 diffusion from the core to the surrounding medium, thereby realizing the continuous release of both VEGF and BMP-2. The aforementioned continuous growth factor release profiles, which mimic their secretion processes in natural bone repair, represent a promising strategy for accelerating the growth factor-driven bone regeneration process [9, 13].

Maintaining the activity of growth factors is another major challenge in the use of microspheres for their delivery. Our results showed that VEGF and BMP-2 released from the PLGA/PLLA core-shell microspheres were able to promote the proliferation of VECs and the osteogenic differentiation of osteoblasts, respectively. VECs specifically targeted by VEGF are usually considered as the intimal layer of new blood vessels and play a key role in angiogenesis. Cells of the MC3T3-E1 line are precursor cells to osteoblasts, and BMP-2 can promote their osteogenesis and differentiation [13]. Therefore, the delivery of VEGF and BMP-2 via core-shell microspheres may promote angiogenesis and osteogenesis in vivo and improve bone formation [22, 23]. In the present study, the maintenance of the biological activity of the growth factors may be attributed to the compatibility of the electrostatic spray method, which involves a step-by-step process without the use of potentially hazardous organic solvents or crosslinking agents.

The biocompatibility of the different formulations of microspheres prepared in this study was investigated in vitro. The results for cell proliferation and viability for both osteoblasts and VECs indicated that all groups of microspheres had no obvious cytotoxicity and possessed good biocompatibility. In addition, the microspheres loaded with BMP-2 in either the core or shell promoted the osteogenic differentiation of the osteoblast precursor cells, while microspheres loaded with only VEGF or no growth factor had no obvious effect on promoting the osteogenic differentiation of these cells. These findings imply that the BMP-2 released from the microspheres played an essential role in promoting bone differentiation, whereas VEGF released from the microspheres only promoted the proliferation of osteoblasts in vitro. Among the microspheres loaded with BMP-2, the BMP-2/VEGF group showed the greatest ability to promote bone differentiation. Considering these microspheres had BMP-2 in the core and VEGF in the shell, our results indicate that the sequential release of VEGF and BMP-2 had a synergistic effect on bone differentiation. This finding is consistent with the research results of Wang et al. [13].

With respect to the effects of growth factor-loaded core-shell microspheres on VECs, all microsphere formulations containing VEGF could promote the proliferation of VECs to some extent, as well as the expression of the angiogenesis-related VEGF and HIF-1*α* genes. The action of VEGF may enhance the effect of BMP-2 in promoting bone differentiation via two mechanisms. Firstly, VEGF can promote the construction of a vascular network, which then indirectly accelerates the formation of new bone [24]. Secondly, VEGF may participate in the BMP signaling pathway and enhance the activity of BMP-2. Previous research has shown that the interaction between VEGF and BMP-2 may promote the nuclear translocation of osterix protein by activating the p38 MAPK pathway, thereby promoting osteogenic differentiation [25].

## 5. Conclusion

The coaxial electrospray technique can be applied to successfully prepare dual growth factor-loaded core-shell microspheres that can achieve the slow sequential release of VEGF and BMP-2. While all prepared core-shell microspheres showed good biocompatibility, the VEGF/BMP-2 core-shell microspheres exhibited the strongest ability to promote bone differentiation along with the proliferation of VECs.

## Figures and Tables

**Table 1:** Preparation of different microspheres.

	Group A	Group B	Group C	Group D	Group E	Group F
Polymer type	PLLA (core)/PLGA (shell)	PLLA (core)/PLGA (shell)	PLLA (core)/PLGA (shell)	PLLA (core)/PLGA (shell)	PLLA (core)/PLGA (shell)	PLLA (core)/PLGA (shell)
Polymer concentration	PLLA (5%)/PLGA (5%)	PLLA (5%)/PLGA (5%)	PLLA (5%)/PLGA (5%)	PLLA (5%)/PLGA (5%)	PLLA (5%)/PLGA (5%)	PLLA (5%)/PLGA (5%)
Growth factor type	BMP-2 (core)/VEGF (shell)	BMP-2 (core)/blank (shell)	Blank (core)/VEGF (shell)	BMP-2, VEGF (core)/BMP-2, VEGF (shell)	VEGF (core)/BMP-2 (shell)	Blank (core)/blank (shell)
Growth factor solution	BMP-2 (100 *μ*g/ml)/VEGF (10 *μ*g/ml)	BMP-2 (100 *μ*g/ml)/blank (shell)	Blank (core)/VEGF (10 *μ*g/m)	BMP-2 (100 *μ*g/ml), VEGF (10 *μ*g/ml)/BMP-2 (100 *μ*g/ml), VEGF (10 *μ*g/ml)	VEGF (10 *μ*g/ml)/BMP-2 (100 *μ*g/ml)	Blank (core)/blank (shell)

**Table 2:** Primer sequences used for detection of osteogenesis-related gene expression.

Gene	Primer	Primer sequence (5′-3′)	Product length (bp)
*Actin*	Forward	ACATCCGTAAAGACCTCTATGCC	223
	Reverse	TACTCCTGCTTGCTGATCCAC	

*RUNX-2*	Forward	CTCCTCTGTCCCGTCACCT	135
	Reverse	ATCACAACAGCCACAAGTTAGCG	

*OCN*	Forward	CTGACCTCACAGATGCCAA	192
	Reverse	CATACTGGTCTGATAGCTCGT	

*OPN*	Forward	GAGGAAACCAGCCAAGGTAA	117
	Reverse	CCAAACAGGCAAAAGCAAAT	

**Table 3:** Primer sequences used for detection of angiogenesis-related gene expression.

Gene	Primer	Primer sequence (5′-3′)	Product length (bp)
*Actin*	Forward	ACATCCGTAAAGACCTCTATGCC	223
	Reverse	TACTCCTGCTTGCTGATCCAC	

*VEGF*	Forward	GAACCAGACCTCTCACCGGAA	135
	Reverse	ACCCAAAGTGCTCCTCGAAG	

*HIF-1α*	Forward	TCCAGCAGACCCAGTTACAGA	182
	Reverse	GCCACTGTATGCTGATGCCTT	

**Table 4:** Encapsulation efficiency for growth factors in microspheres.

	BMP-2/VEGF group	BMP-2/Blank group	Blank/VEGF group	Double/Double group	VEGF/BMP-2 group
Encapsulation efficiency (%)					
VEGF	73.2 ± 2.7	—	70.2 ± 3.3	71.5 ± 2.1	83.1 ± 1.9
BMP-2	84.1 ± 3.2	85.7 ± 2.6	—	80.3 ± 4.7	74.2 ± 2.3

## Data Availability

The data that support the findings of this study are available from the corresponding authors upon reasonable request.

## References

[1] Yu X., Tang X., Gohil S. V., Laurencin C. T. (2015). Biomaterials for bone regenerative engineering. *Advanced Healthcare Materials*.

[2] Pearlin, Nayak S., Manivasagam G., Sen D. (2018). Progress of regenerative therapy in orthopedics. *Current Osteoporosis Reports*.

[3] Mercado-Pagán Á. E., Stahl A. M., Shanjani Y., Yang Y. (2015). Vascularization in bone tissue engineering constructs. *Annals of Biomedical Engineering*.

[4] Atienza-Roca P., Cui X., Hooper G. J., Woodfield T. B. F., Lim K. S. (2018). Growth factor delivery systems for tissue engineering and regenerative medicine. *Advances in Experimental Medicine and Biology*.

[5] Lim K. S., Baptista M., Moon S., Woodfield T. B. F., Rnjak-Kovacina J. (2019). Microchannels in development, survival, and vascularisation of tissue analogues for regenerative medicine. *Trends in Biotechnology*.

[6] De Matos M. B. C., Piedade A. P., Alvarez-Lorenzo C., Concheiro A., Braga M. E. M., De Sousa H. C. (2013). Dexamethasone-loaded poly(ɛ-caprolactone)/silica nanoparticles composites prepared by supercritical CO_2_ foaming/mixing and deposition. *International Journal of Pharmaceutics*.

[7] Amini N., Mazinani S., Ranaei-Siadat S. O. (2013). Acetylcholinesterase immobilization on polyacrylamide/functionalized multi-walled carbon nanotube nanocomposite nanofibrous membrane. *Applied Biochemistry and Biotechnology*.

[8] Yang X. L., Ju X. J., Mu X. T. (2016). Core–shell chitosan microcapsules for programmed sequential drug release. *ACS Applied Materials & Interfaces*.

[9] Mehta M., Schmidt-Bleek K., Duda G. N., Mooney D. J. (2012). Biomaterial delivery of morphogens to mimic the natural healing cascade in bone. *Advanced Drug Delivery Reviews*.

[10] Wang Y., Wei Y., Zhang X. (2015). PLGA/PDLLA core-shell submicron spheres sequential release system: preparation, characterization and promotion of bone regeneration in vitro and in vivo. *Chemical Engineering Journal*.

[11] Kusumbe A. P., Ramasamy S. K., Adams R. H. (2014). Coupling of angiogenesis and osteogenesis by a specific vessel subtype in bone. *Nature*.

[12] Hasegawa T., Tsuchiya E., Abe M., Amizuka N. (2016). Cellular interplay of bone cells and vascular endothelial cells in bone. *Clinical Calcium*.

[13] Wang Q., Zhang Y., Li B., Chen L. (2017). Controlled dual delivery of low doses of BMP-2 and VEGF in a silk fibroin-nanohydroxyapatite scaffold for vascularized bone regeneration. *Journal of Materials Chemistry B*.

[14] Ghaffarzadegan R., Khoee S., Rezazadeh S. (2020). Fabrication, characterization and optimization of berberine-loaded PLA nanoparticles using coaxial electrospray for sustained drug release. *Daru*.

[15] Chen C., Liu W., Jiang P., Hong T. (2019). Coaxial electrohydrodynamic atomization for the production of drug-loaded micro/nanoparticles. *Micromachines*.

[16] Chang M. W., Stride E., Edirisinghe M. (2010). A new method for the preparation of monoporous hollow microspheres. *Langmuir*.

[17] Choi D. H., Subbiah R., Kim I. H., Han D. K., Park K. (2013). Dual growth factor delivery using biocompatible core–shell microcapsules for angiogenesis. *Small*.

[18] Nie H., Dong Z., Arifin D. Y., Hu Y., Wang C. H. (2010). Core/shell microspheres via coaxial electrohydrodynamic atomization for sequential and parallel release of drugs. *Journal of Biomedical Materials Research Part A*.

[19] Juhl O., Zhao N., Merife A. B. (2019). Aptamer-functionalized fibrin hydrogel improves vascular endothelial growth factor release kinetics and enhances angiogenesis and osteogenesis in critically sized cranial defects. *ACS Biomaterials Science & Engineering*.

[20] Perri B., Cooper M., Lauryssen C., Anand N. (2007). Adverse swelling associated with use of rh-BMP-2 in anterior cervical discectomy and fusion: a case study. *The Spine Journal*.

[21] Kempen D. H., Lu L., Heijink A. (2009). Effect of local sequential VEGF and BMP-2 delivery on ectopic and orthotopic bone regeneration. *Biomaterials*.

[22] Dou D. D., Zhou G., Liu H. W. (2019). Sequential releasing of VEGF and BMP-2 in hydroxyapatite collagen scaffolds for bone tissue engineering: design and characterization. *International Journal of Biological Macromolecules*.

[23] Zhang H. X., Zhang X. P., Xiao G. Y. (2016). *In vitro* and *in vivo* evaluation of calcium phosphate composite scaffolds containing BMP-VEGF loaded PLGA microspheres for the treatment of avascular necrosis of the femoral head. *Materials Science and Engineering: C*.

[24] Subbiah R., Hwang M. P., Van S. Y. (2015). Osteogenic/angiogenic dual growth factor delivery microcapsules for regeneration of vascularized bone tissue. *Advanced Healthcare Materials*.

[25] Wang T., Guo S., Zhang H. (2018). Synergistic effects of controlled-released BMP-2 and VEGF from nHAC/PLGAs scaffold on osteogenesis. *BioMed Research International*.

